# Influence of Hyperoxic-Supplemented High-Intensity Interval Training on Hemotological and Muscle Mitochondrial Adaptations in Trained Cyclists

**DOI:** 10.3389/fphys.2019.00730

**Published:** 2019-06-14

**Authors:** D. A. Cardinale, F. J. Larsen, J. Lännerström, T. Manselin, O. Södergård, S. Mijwel, P. Lindholm, B. Ekblom, R. Boushel

**Affiliations:** ^1^Åstrand Laboratory, The Swedish School of Sport and Health Sciences, Stockholm, Sweden; ^2^Department of Physiology and Pharmacology, Karolinska Institutet, Stockholm, Sweden; ^3^School of Kinesiology, Faculty of Education, University of British Columbia, Vancouver, BC, Canada

**Keywords:** cycling performance, hyperoxia, high-intensity interval training, mitochondria, OXPHOS, VO_2_max

## Abstract

**Background:** Hyperoxia (HYPER) increases O_2_ carrying capacity resulting in a higher O_2_ delivery to the working muscles during exercise. Several lines of evidence indicate that lactate metabolism, power output, and endurance are improved by HYPER compared to normoxia (NORM). Since HYPER enables a higher exercise power output compared to NORM and considering the O_2_ delivery limitation at exercise intensities near to maximum, we hypothesized that hyperoxic-supplemented high-intensity interval training (HIIT) would upregulate muscle mitochondrial oxidative capacity and enhance endurance cycling performance compared to training in normoxia.

**Methods:** 23 trained cyclists, age 35.3 ± 6.4 years, body mass 75.2 ± 9.6 kg, height 179.8 ± 7.9 m, and VO_2_max 4.5 ± 0.7 L min^−1^ performed 6 weeks polarized and periodized endurance training on a cycle ergometer consisting of supervised HIIT sessions 3 days/week and additional low-intensity training 2 days/week. Participants were randomly assigned to either HYPER (F_I_O_2_ 0.30; *n* = 12) or NORM (F_I_O_2_ 0.21; *n* = 11) breathing condition during HIIT. Mitochondrial respiration in permeabilized fibers and isolated mitochondria together with maximal and submaximal VO_2_, hematological parameters, and self-paced endurance cycling performance were tested pre- and posttraining intervention.

**Results:** Hyperoxic training led to a small, non-significant change in performance compared to normoxic training (HYPER 6.0 ± 3.7%, NORM 2.4 ± 5.0%; *p* = 0.073, ES = 0.32). This small, beneficial effect on the self-paced endurance cycling performance was not explained by the change in VO_2_max (HYPER 1.1 ± 3.8%, NORM 0.0 ± 3.7%; *p* = 0.55, ES = 0.08), blood volume and hemoglobin mass, mitochondrial oxidative phosphorylation capacity (permeabilized fibers: HYPER 27.3 ± 46.0%, NORM 16.5 ± 49.1%; *p* = 0.37, ES = 3.24 and in isolated mitochondria: HYPER 26.1 ± 80.1%, NORM 15.9 ± 73.3%; *p* = 0.66, ES = 0.51), or markers of mitochondrial content which were similar between groups post intervention.

**Conclusions:** This study showed that 6 weeks hyperoxic-supplemented HIIT led to marginal gain in cycle performance in already trained cyclists without change in VO_2_max, blood volume, hemoglobin mass, mitochondrial oxidative phosphorylation capacity, or exercise efficiency. The underlying mechanisms for the potentially meaningful performance effects of hyperoxia training remain unexplained and may raise ethical questions for elite sport.

## Introduction

It is still under the debate whether strategies such as hyperoxia supplementation enabling acute improvement of exercise performance leads to superior training adaptations and therefore greater performance enhancement (Ploutz-Snyder et al., 1996; Hamalainen et al., 2000; Morris et al., 2000; Perry et al., 2005, 2007; Kilding et al., 2012; Przyklenk et al., 2017). However, despite the fact that previous studies have employed different study designs, training intervention lengths (3–6 weeks), exercise mode (continuous or high-intensity interval training), fractions of O_2_ inspired (F_I_O_2_ range: 0.26–0.70), training status of the participants (i.e., untrained or trained cyclists), and different physical performance tests (all-out effort or time to exhaustion), an overall “likely positive” effect on performance has been found following hyperoxic-supplemented training compared to normoxic training (Mallette et al., 2017; Cardinale and Ekblom, 2018).

As recently reviewed (Cardinale and Ekblom, 2018), hyperoxia acutely improves lactate metabolism (Ekblom et al., 1975), reduces muscle glycogen utilization (Stellingwerff et al., 2006), and enables a higher exercise work rate compared to exercise in normoxia (Powers et al., 1989; Nielsen et al., 1999) while reducing submaximal exercise efficiency (Manselin et al., 2017). Acutely, hyperoxia increases oxygen (O_2_) delivery to the working muscles (Ekblom et al., 1975) and completely prevents exercise-induced arterial hypoxemia (EIAH), i.e., “oxyhemoglobin SaO_2_ below 95%” (Powers et al., 1989), a condition often found in individuals exercising at intensities approaching maximal oxygen uptake (VO_2_max) (Dempsey and Wagner, 1999; Dempsey et al., 2008). Considering the O_2_ delivery limitation at near maximal exercise intensities (Saltin and Calbet, 2006) and that the mitochondria possess an excess capacity above the O_2_ delivery (Boushel et al., 2011), we postulated that hyperoxic-supplemented exercise training allowing a higher training load leads to a greater training stimulus at the muscle level and therefore greater performance enhancement compared to the same training regimen breathing normoxia. This hypothesis is supported by the finding that exercise training with recombinant human erythropoietin treatment enhanced skeletal muscle mitochondrial capacity compared to controls (Plenge et al., 2012).

Only a few studies have assessed markers of skeletal muscle metabolic adaptions following hyperoxic-supplemented endurance training (Ploutz-Snyder et al., 1996; Perry et al., 2007; Przyklenk et al., 2017). The greater enzyme activity involved in β-oxidation following hyperoxic training indicated by the higher β-hydroxyacyl-coenzyme A dehydrogenase shown by Ploutz-Snyder et al. (1996), was not later found by Perry et al. (2007). In both studies, glycolytic enzymes activities (phosphofructokinase, creatine kinase, and glyceraldehyde phosphate dehydrogenase) and components of oxidative phosphorylation (cytochrome c-oxidase and citrate synthase) were not enhanced with hyperoxic training. Furthermore, previous studies have been conducted on untrained or recreationally trained individuals, and the mechanism of action of hyperoxic-supplemented endurance training may differ in trained individuals. Only two studies have been conducted on trained individuals (Hamalainen et al., 2000; Kilding et al., 2012) with opposite results on performance, and without detail on potential physiological mechanisms. For these reasons, we designed a 6-week randomized controlled training study with parallel groups where both participants and training supervisors were blinded to the type of gas inhaled with the aim to assess the effect of hyperoxic-supplemented high-intensity interval training on physiological and performance outcomes in trained cyclists.

We hypothesized that hyperoxic-supplemented high-intensity interval training in trained cyclists would enhance the training stimulus on skeletal muscle and thereby improve cycle performance to a greater extent than a normoxic breathing condition due to an upregulated skeletal muscle mitochondrial oxidative phosphorylation capacity independent of cardiorespiratory or hematological adaptations.

## Materials and Methods

### General Design

This study used a 6-week double-blind randomized controlled training study design with parallel groups stratified for participants’ baseline VO_2_max. Participants were randomly assigned to either an experimental group that trained breathing hyperoxia (F_I_O_2_ 0.30; HYPER) or a sham-hyperoxia control group that trained in normoxia (F_I_O_2_ 0.21; NORM). Within a week prior to and posttraining intervention, participants reported to the laboratory three times for baseline and post-tests, respectively. The training intervention consisted of 15 supervised high-intensity interval training (HIIT) bouts breathing either HYPER or NORM and 10 non-supervised low-intensity exercise sessions distributed within a 6-week period (explained in detail in “the training intervention” section). Inclusion criteria in the final analysis were an attendance of at least 85% of the HIIT and low-intensity training sessions. Participants were informed of the possible risks and discomfort involved before giving their written consent to participate. The study was undertaken according to the Declaration of Helsinki and was approved by the Swedish Regional Ethics Committee (2014/1764-31/2 and 2017/630-32).

### Participants

A group of 32 trained cyclists (24 men and 7 women), age 34.8 ± 7.3 years [mean ± standard deviation (SD)], body mass 72.9 ± 10.8 kg, height 177.7 ± 9.6 m, and VO_2_max 4.4 ± 0.8 L min^−1^ participated in this study. Prior to inclusion, a larger group of cyclists completed a health screening survey and exercise tests for assessment of their cardiorespiratory fitness. Only the healthy subjects who regularly conducted endurance and ultra-endurance races and had been competing at a national or amateur level in the last 5–10 years prior to this study were selected for this study. The aim of including experienced cyclists is that this group of athletes shows a small magnitude of change in performance, cardiorespiratory fitness as well as metabolic adaptation even when intensifying their training. On average, each subject was accustomed to about 10–15 h exercise training per week. High-intensity interval training and resistance training was consistently implemented in their normal training regimen on average once per week; however 80–90% of the whole training time was spent at low and average exercise intensity.

### Training Intervention

The training intervention consisted of a 6-week polarized and periodized endurance training on a cycle ergometer. The term *polarized* refers to the combination of both HIIT and low-intensity exercise sessions within the same mesocycle (i.e., 6 weeks) (Stöggl and Sperlich, 2014), whereas the term *periodized* refers to the variation of training load and intensity within the mesocycle. The 6-week intervention protocol used a periodization model with a relationship of 2:1 between hard weeks and easy weeks. This approach was taken to reduce the risk of overtraining syndrome and included a tapering period of a week before the post-tests assessments. Participants were scheduled to perform 15 supervised HIIT sessions breathing either HYPER or NORM and 10 low-intensity exercise sessions.

The HIIT sessions, scheduled on Monday, Wednesday and Friday each week, consisted of either three times 8-min intervals or four times 4-min intervals performed at maximal sustainable effort [equivalent to a rating perceived exertion, RPE, of 18–20 (Borg, 1970)], with 3-min active relief in between the exercise bouts on a cycle ergometer. The longer intervals were performed three times per week during weeks 1, 2, 4, and 5 of the training intervention, whereas the 4-by-4-min intervals were performed twice in week 3 and once in week 6. To progressively increase the participants’ training load during the training intervention, a fourth interval of 4 min in length in week 4 and a fourth interval of 8 min in length in week 5 was added, in addition to the three times 8-min intervals as described above.

Each HIIT session was preceded by a standard 20-min warm up protocol on a cycle ergometer which included two bouts of 1 min at moderate/high-intensity exercise. This type of warm up has been shown to be superior to continuous warm up either at an intensity below or above threshold (McGowan et al., 2015). Overall, the 20-min warm up was performed as follows: 10 min cycling at an exercise intensity equivalent to 10–12 RPE, 1 min at 14–16 RPE, 3 min at 11–14 RPE, 1 min at 15–17 RPE, and 5 min at 10–12 RPE. During the last 30 s of the warm-up protocol, the participants wore a full-face mask with headgear used for gas administration (refer to section “Gas Administration” for the complete setup description). The participants removed the mask during the relief periods. At the completion of the HIIT session, a 10-min cool-down was performed at an exercise intensity equivalent to 10–12 RPE.

During the HIIT sessions, the only data shown to the participants were the cycling cadence and the elapsed exercise time, whereas the participants were blinded to breathing condition, power output, and heart rate. Since the HIIT sessions were self-paced by the participants who were blinded to power output, one or two training supervisors guided and encouraged the participants to assure that each interval of each session was performed at the highest sustainable intensity possible by the participants. For motivational and time-efficiency purposes, participants completed HIIT sessions in the company of 2–4 other participants. The training supervisors were also blinded to the breathing condition that the participants were assigned to during the whole duration of the training intervention.

Participants were encouraged to perform two low-intensity exercise training sessions consisting of about 2 h (LOW1) and 4 h (LOW2) long continuous exercise at ~75% of the individual maximal heart rate (equivalent to an RPE of 14–15). The participants conducted the LOW1 cycling either outdoors or on a stationary bike indoors on Tuesday or Thursday of weeks 1, 2, 4, 5, and 6 of the training intervention. A LOW2 session was scheduled each weekend during the 6-week training intervention.

Participants were requested to maintain supplementary training involving flexibility exercise and exercise specifically recruiting upper body muscles. No other strenuous exercise training was allowed during the 6-week intervention except for the one above described.

### Gas Administration

The gas was delivered to the participants through a face mask which was connected, by tubing, to a dosage unit (Oxelerate, Tumba, Sweden) which in turn was connected to a gas tank filled with either pure medical oxygen or medical air gas (i.e., 21% O_2_). For subjects assigned to the hyperoxia group, the dosage unit intermittently delivered a gas bolus at the beginning of each participant’s inhalation that was mixed with room air inside the mask cavity resulting in a final F_I_O_2_ of ~0.30, as established by previous work (Lindholm et al., 2017). The participants and the training supervisors were blinded to the type of gas inhaled.

### Procedures and Measures

Participants reported to the laboratory on three separate occasions with 2–3 days in between within the week prior to the start of the exercise intervention and a week after the last training session. Participants abstained from strenuous physical activity 24–48 h prior to each occasion.

The first occasion consisted of skeletal muscle biopsy collection and total hemoglobin mass assessment which was scheduled in the morning between 07:00 and 10:00 h to limit the circadian influence. At the second occasion, participants performed a submaximal and maximal incremental test on cycle ergometer in the afternoon between 15:00 and 19:00 h. The third occasion consisted of a self-paced cycling performance test scheduled between 09:00 and 17:00 h.

#### Muscle Biopsy Sampling

A skeletal muscle sample was obtained from the middle portion of the *vastus lateralis* muscle at a depth of 2–3 cm, about one-third of the distance from the upper margin of the patella to the anterior superior iliac spine with the participants resting in a semi recumbent position lying on a bench. After local anesthesia (2–4 ml carbocaine 20 mg ml^−1^; AstraZeneca, Södertälje, Sweden), an incision (0.5–1 cm) was made through the skin and fascia and a muscle sample (50–100 mg) was obtained with the Weil-Blakesley chonchotome technique. A portion of the sample was snap frozen in liquid nitrogen, while second and third portions were rapidly placed in ice-cold mitochondrial isolation medium and relaxing medium (see section below), followed by mitochondria isolation and muscle fiber permeabilization, respectively, as later described.

#### Hematological Parameters

A subgroup of 12 participants (*n* = 6 per group) was tested for total hemoglobin mass (Hb_mass_) assessment performed as described elsewhere (Burge and Skinner, 1995) with some minor modification of the rebreathing technique. Briefly, with the participants still lying on the bench in a semi recumbent position following the biopsy collection, 15 ml of blood was sampled from an antecubital vein *via* a 20-gauge venflon and analyzed immediately for Hb concentration (Hb) using HemoCue® Hb 201+ System (HemoCue AB, Ängelholm, Sweden); and hematocrit in quadruplets with micro-method (3 min at 13,500 rpm). About 1.5 ml of the blood sample was then quickly transferred to a 2-ml Eppendorf tube and stored at −80°C until percent carboxyhemoglobin (%HbCO) analysis using an hemoximeter (ABL800, Radiometer, Copenhagen, Denmark). After baseline collection, the participants breathed from a Douglas bag previously filled with pure oxygen for 4 min to flush nitrogen from the airways. During this time, the operator flushed the re-breathing circuit with the pure oxygen which was then closed. After 4 min, the operator switched the participant to the rebreathing circuit and a precisely measured bolus of 1.2 ml kg^−1^ body mass of 99.997% chemically pure CO (CO N47, Air Liquide, Paris, France) was injected into the circuit. The participants then breathed the gas mixture for 10 min. Thereafter, an additional venous blood sample was collected from an antecubital vein for assessment of the change in (%HbCO) accounting for the CO remaining in the re-breathing circuit which was determined (Monoxor III, Bacharach Inc., New Kensington, USA). Hb_mass_ was calculated from the change in %HbCO and total red blood cell volume (RCV), blood volume (BV), and plasma volume (PV) were derived (Burge and Skinner, 1995).

#### Submaximal Exercise Test

To determine the power output at lactate inflection point using a modified Dmax method, a sub-group of 12 participants (*n* = 6 per group) cycled at 4–6 intervals (i.e., 30, 150, 185, 220, 255, 290, and 325 W) each of 4 min in length on a cycle ergometer (Monark LC6, Monark Exercise AB, Vansbro, Sweden). Participants cycled with a constant, freely chosen cadence until blood lactate concentration (Biosen C-Line Clinic; EKF-diagnostic GmbH, Barleben, Germany) measured at the end of each interval from fingertips, reached a concentration higher than 4 mMol L^−1^. Participants cycled wearing a Hans Rudolf mask (Hans Rudolph Inc., Kansas, USA) which covered mouth and nose for assessment of pulmonary oxygen consumption (Jaeger Oxycon Pro; CareFusion GmbH, Hoechberg, Germany). The metabolic cart was calibrated prior to each test according to the manufacturer’s instructions, with high-grade calibration gases (Air Liquide, Paris, France). Respiratory variables were measured and averaged every 10 s. The averaged VO_2_ recorded during the last minute of each interval was taken as the representative oxygen consumption for that specific power output. After the last interval, participants were allowed 10 min cycling at an intensity equal to 10–12 RPE to recover and get ready for subsequent graded incremental exercise test.

The blood lactate concentrations representative for each exercise intensity were used to calculate the power output at lactate inflection point using a modified Dmax method (Cheng et al., 1992), i.e., defined as the derivate to the exponential curve created from exponential lactate increase, including maximal lactate concentration obtained from the successive maximal increment test. The increase in lactate relative to power output was defined as the increase in blood lactate from the point where the exponential curve crossed the lactate baseline.

The mean oxygen consumption and the respiratory exchange ratio between minute 3 and 4 at each cycled stage was used to calculate energy expenditure with the equation developed by Brouwer (1957). Cycling efficiency when cycling at 150 W was expressed as gross efficiency (GE) and work efficiency (WE) as described elsewhere (Mogensen et al., 2006).

#### Maximal Incremental Test

All participants performed a graded incremental exercise test until volitional exhaustion on a cycle ergometer to determine VO_2_max and time to exhaustion. Participants pedaled at a fixed cadence and the load was increased by 25 and 20 W min^−1^ for men and women, respectively. Participants cycled wearing a Hans Rudolf mask (Hans Rudolph Inc., Kansas, USA) which covered mouth and nose for assessment of pulmonary oxygen consumption (Jaeger Oxycon Pro; CareFusion GmbH, Hoechberg, Germany). VO_2_max leveling-off criteria were applied (i.e., a VO_2_ plateau, followed by exercise cessation or decrease of VO_2_ at higher work rates, with an RER >1.10). The power output that the participants pedaled at the time of the volitional exhaustion was taken as the maximal power output (Winc.). O_2_ consumption was measured with a metabolic cart (OxyconPro, Jaeger GmbH, Germany), calibrated prior to each test according to the manufacturer’s instructions, with high-grade calibration gases (Air Liquide, Paris, France). Respiratory variables were measured and averaged every 10 s. The highest 60 s averaged VO_2_ recorded was taken as the VO_2_max.

#### Cycle Performance Test

The self-paced endurance cycling performance test consisted of pedaling for 20 min at the maximal sustainable effort with the intent to obtain the highest mean power output on a cycle ergometer (Monark LC2, Monark Exercise AB, Vansbro, Sweden) equipped with a power meter (SRM power meter science road; SRM International, Jülich, Germany) for power output assessment. The test was preceded by a standard 20-min warmup as described in the “Training intervention” section. Briefly, the participants cycled for 10 min at an exercise intensity equivalent to 10–12 RPE, 1 min at 14–16 RPE, 3 min at 11–14 RPE, 1 min at 15–17 RPE, and 5 min at 10–12 RPE. During the last 5 min of the warm up, the participants were equipped with a Hans Rudolf mask (Hans Rudolph Inc., Kansas, USA) which covered mouth and nose for assessment of pulmonary oxygen consumption (Jaeger Oxycon Pro; CareFusion GmbH, Hoechberg, Germany) and a pulse oximetry sensor (Rad-97 Masimo Corporation; Neuchatel, Switzerland) was positioned on the participant’s forehead for assessment of the peripheral capillary oxygen saturation (SpO_2_).

During the test, the only data shown to participants were the cycling cadence and the elapsed exercise time, whereas the participants were blinded to power output and heart rate. The test leader verbally encouraged the participants during the whole test length. For motivational purposes, music chosen by the participant was played during test. The participants completed the test with a 10 min cool-down performed at an exercise intensity equivalent to 10–12 RPE. Participants were all familiar to this test and performed one or two familiarization tests prior the baseline test, which showed a typical error of measurement of about 2%.

#### Permeabilized Fiber Preparation

Muscle fiber bundle permeabilization was performed as previously described (Pesta and Gnaiger, 2012; Cardinale et al., 2018a). Briefly, a portion of the muscle biopsy (~5 mg wet weight) was immediately transferred into ice-cold relaxing medium (BIOPS) containing 10 mM/L Ca^2+^/EGTA buffer, 20 mM/L imidazole, 50 mM/L K^+^-4-morpholinoethanesulfonic acid (Mes), 0.5 mMol/L dithiothreitol, 6.56 mM/L MgCl_2_, 5.77 mM/L ATP, and 15 mMol/L phosphocreatine at pH 7.1. A portion of the 5 mg wet weight sample (~1–3 mg) was transferred into BIOPS in a small petri dish on an ice-cold metal plate where the fiber bundles were mechanically separated using forceps and needles. Thereafter, approximately 10–15 fibers were slowly agitated for 30 min on a platform shaker in BIOPS containing saponin (5 mg/ml saponin) solution at 4°C. Fibers were then washed for 10 min at 4°C in ice-cold mitochondrial respiration medium (MiR06; 0.5 mM EGTA, 3 mM MgCl2, 60 mM K-lactobionate, 20 mM taurine, 10 mM KH_2_PO_4_, 20 mM HEPES, 110 mM sucrose, and 1 g/L BSA essentially fatty acid free, adjusted to pH 7.1, 2.8 U/mg solid catalase lypophilized powder). The fibers were weighed on a microbalance after having been blotted on filter paper and transferred into the respirometry chamber.

#### Isolation of Mitochondria

A portion of the muscle biopsy (40–80 mg wet weight) designated for mitochondrial isolation was first weighed and then cut in ice-cold isolation medium (sucrose 100 mM, KCl 100 mM, Tris-HCl 50 mM, KH_2_PO_4_ 1 mM, EGTA 100 μM, BSA 0.1%; final pH was set to 7.4). The homogenate was washed in 1 ml isolation medium and the supernatant was removed. One ml of isolation medium containing 0.2 mg ml^−1^ bacterial protease was added to the homogenate. The homogenate was gently agitated every 30 s for ~2-min time and then transferred in a pre-cooled glass jacket connected to an ice-cold bath pump and further homogenized with a hand held electrically driven drill (80 rpm). The final homogenate was then transferred to a falcon tube containing 3 ml isolation medium and then subsequently centrifuged at 700 *g* at 4°C for 10 min. After removing the pellet, the suspension was again centrifuged at 10,000 *g* at 4°C. The resultant mitochondrial pellet was re-suspended in the same medium. The Eppendorf was then centrifuged at 7,000 *g* for 5 min and the pellet was dissolved in 0.6 μl preservation medium (EGTA 0.5 mM, MgCl_2_⋅6H_2_O 3 mM, K-lactobionate 60 mM, Taurine 20 mM, KH_2_PO_4_ 10 mM, HEPES 20 mM, sucrose 110 mM, BSA 1 g L^−1^ histidine 20 mM, vitamin E succinate 20 μM, glutathione 3 mM, leupeptine 1 μM, glutamate 2 mM, malate 2 mM, and Mg-ATP 2 mM) per mg wet weight.

#### Mitochondrial Respiration

Mitochondrial respiration was performed in a two-channel high-resolution respirometer (Oroboros Oxygraph, Paar, Graz, Austria). The glass chamber volume (2 ml capacity) was sealed with rubber-ringed stoppers to minimize the O_2_ back diffusion into the chamber (Steinlechner-Maran et al., 1996). Polyvinylidene difluoride magnetic stirrers set to 750 rpm were used to stir the sample in to the medium. Data were collected at 1-s intervals and averaged over 40 s. All experiments were run in duplicate and the respiration data for each of the two chambers were then averaged. The medium in the respiration chamber was MiR05 containing EGTA 0.5 mM, MgCl_2_⋅6H_2_O 3 mM, K-lactobionate 60 mM, taurine 20 mM, KH_2_PO_4_ 10 mM, HEPES 20 mM, sucrose 110 mM, and BSA 1 g L^−1^. All experiments were performed at 37°C. Time constants for complete mixing in the chamber were calculated by briefly stopping and starting the stirrers. O_2_ consumption and zero-drift of the O_2_ electrode were calculated using DatLab 5.2 software (Oroboros, Paar, Graz, Austria). At least five different O_2_ tensions (400–0 nMol/ml) were used during the background calibration to calculate and account for the diffusion of O_2_ into the chamber.

#### Mitochondrial Respiration in Permeabilized Fibers

Mitochondrial respiration was measured by adding the following substrates into the chambers (final concentrations): octanoylcarnitine (0.2 mM), malate (2 mM) for assessment of leak respiration (EFT_L_), ADP (2.5 mM) to support electron entry from fatty acid β-oxidation through electron-transferring flavoprotein and complex I (EFT_P_), followed by pyruvate (5 mM) and glutamate (10 mM) to stimulate complex I (CI_P_), and succinate (10 mM) to stimulate complex I and II linked respiration (CI + II_P_). Following inhibition of complex III with antimycin A (mM), N,N,N′,N′-tetramethyl-p-phenylenediamine dihydrochloride (0.5 mM) and ascorbate (2 mM) were added followed by sodium azide (100 mM) for cytochrome C oxidase activity assessment. O_2_ flux from the permeabilized fiber preparation was normalized per initial fiber wet weight.

#### Mitochondrial Respiration in Isolated Mitochondria

The same titration protocol described above was used to measure O_2_ flux in isolated mitochondria with the exception that ADP was left to be completely phosphorylated to ATP following β-oxidation respiration allowing respiration proceed to state 4 (S4). Furthermore, carbonyl cyanide m-chloro phenyl hydrazine (0.05 μM steps) was used to measure maximal uncoupled oxidative phosphorylation (Unc). O_2_ flux from isolated mitochondria was normalized by protein concentrations determined in aliquots of supernatant diluted 1:10 in distilled water using the Pierce 660 nm protein assay (Thermo Scientific).

#### Citrate Synthase Activity

A portion of the freeze-dried muscle samples was first cleansed of visible blood, fat and connective tissue and subsequently homogenized in ice-cold buffer (100 μl/mg dry weight) consisting of 50 mM KH_2_PO_4_, 1 mM EDTA and 0.05% Triton X-100 using a Bullet Blender (NextAdvance, Averill Park, NY) with 0.5 mm ZrO beads. The Eppendorf tubes containing the homogenates were rotated for 30 min at 4°C before being centrifuged at 1,400 *g* for 1 min at 4°C. CS activity was measured on a 96-well plate in a reagent solution (in mM): 50 Tris-HCl, 0.2 DTNB, and 30 acetyl-CoA. The reaction was initiated by adding oxaloacetate (10 mM) and the change in absorbance at 412 nm was measured spectrophotometrically at 25°C.

#### Protein Extraction and Immunoblot Analysis

A portion of the snap frozen biopsy sample was (1) freeze-dried, (2) cleansed of visible blood, fat, and connective tissue and subsequently, and (3) homogenized in ice-cold buffer (100 μl/mg dry weight) consisting of 2 mM HEPES (pH 7.4), 1 mM EDTA, 5 mM EGTA, 10 mM MgCl_2_, 1% Triton X-100, 2 mM dithiothreitol, and 1.5% phosphatase and protease inhibitor cocktail (Halt™, Thermo Scientific, Rockford, MD) using a Bullet Blender (NextAdvance, Averill Park, NY) with 0.5 mm ZrO beads. The Eppendorf tubes containing the homogenates were 360° rotated for 30 min at 4°C before being centrifuged at 10,000 *g* for 10 min at 4°C. The obtained supernatant was stored at −80°C. Protein concentrations of the homogenates were determined using the Pierce 660 nm protein assay (Thermo Scientific). Muscle homogenates were diluted with 4× Laemmli sample buffer (Bio-Rad, Richmond, CA) and homogenizing buffer to obtain a final protein concentration that was similar between all samples. Subsequently, all samples were heated at 95°C for 5 min to denature proteins, and then stored at −20°C until further analysis.

Samples were separated by SDS polyacrylamide gel electrophoresis (PAGE) on 26-well Criterion TGX gradient gels (4–20% acrylamide; Bio-Rad). Samples from all three groups were loaded on the same gel. The blots were quantified using Quantity One software version 4.6.3 (BIORAD). To control for appropriate loading and transfer, target proteins were expressed relative to total protein stained at ~95 kDa obtained by staining the membranes with MemCode Reversible Protein Stain Kit (Thermo Scientific) (Antharavally et al., 2004).

The monoclonal primary cytochrome c oxidase antibody (#4850; 1:1,000; Cell Signaling Technology, Danvers, USA) conjugated with a secondary anti-rabbit antibody (#7074; 1:10,000; Cell Signaling Technology, Danvers, USA) was used for the detection of target total protein.

### Statistical Analysis

Normal distribution of the data was checked by assessing skewness. Baseline characteristics of each group were summarized using descriptive statistics. Exact *χ*^2^ tests were used to evaluate if differences existed between groups for categorical variables at baseline. For between-groups analyses, one-way analysis of variance (one-way ANOVA) was conducted using the variable changes pre to post intervention. A two-tailed *p* < 0.05 was considered significant. Analysis of covariance (ANCOVA) with baseline-test results as a covariate and the post-test as the dependent variable was also performed on the same dataset to ensure that baseline measurements did not affect the statistical results. The use of one-way ANOVA or ANCOVA did not influence the interpretation of the study results. Effect sizes and associated confidence intervals were interpreted according to Cohen’s guidelines (Cohen, 1988), effect sizes with scores of 0.2–0.5, 0.5–0.8, and >0.8 were considered small, medium, and large effects, respectively. Paired sample *t*-test was used to assess within-group differences pre- to post measurement. Statistical analyses were carried out using SPSS statistical software version 21 (SPSS Inc., Chicago, Illinois, USA).

## Results

### Training Adherence, Exercise Intensity, and Training Load

**Figure 1:**
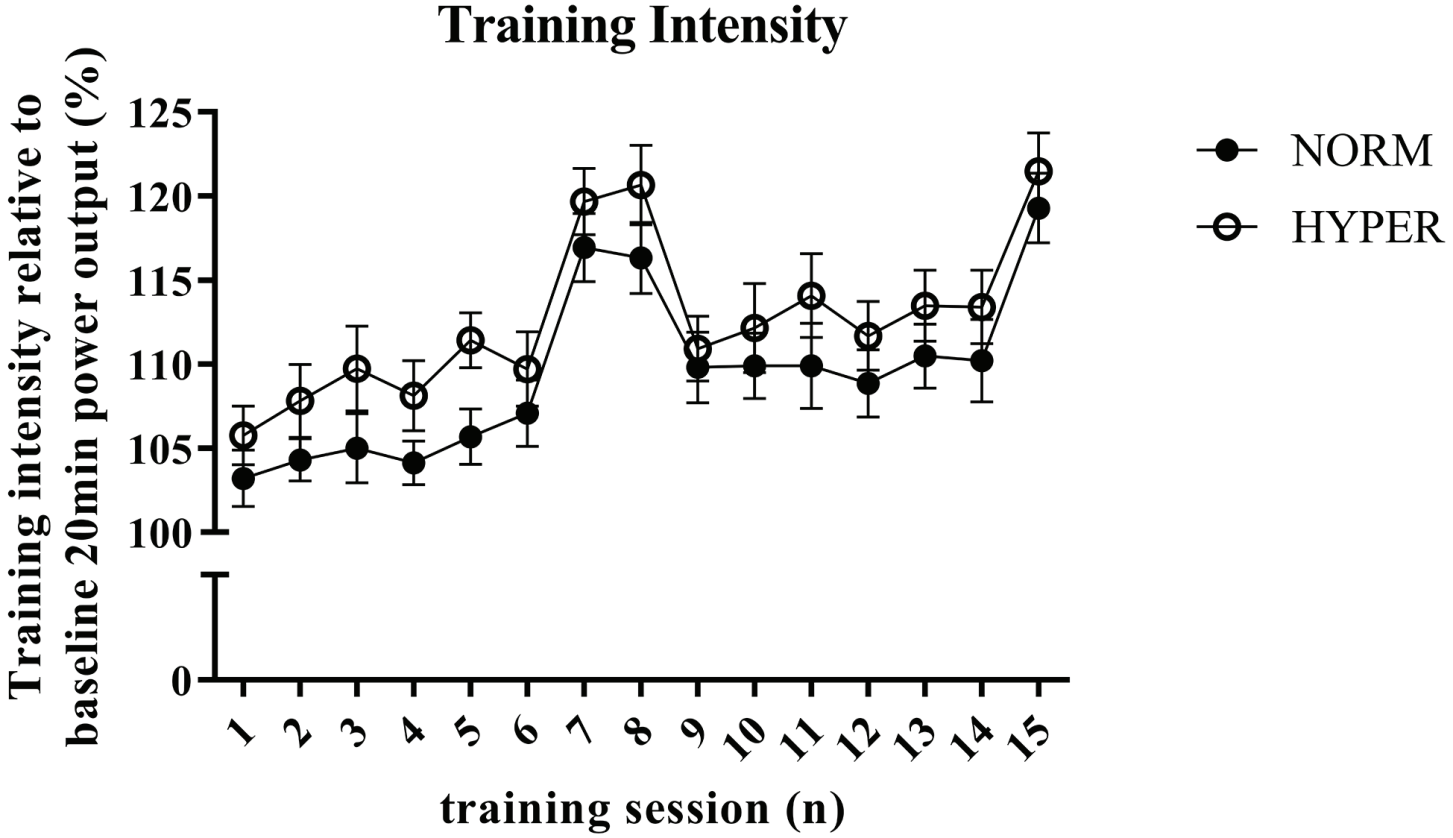
Overview of the training intensity and progression of the normoxic (NORM) and hyperoxic (HYPER) group during the 6-week training intervention. Training intensity of each high-intensity interval session is expressed in percentage of the mean power output obtained during the baseline self-paced 20-min cycling trial. Note that the higher training intensity during training session number 7, 8, and 15 is attributed to the higher exercise intensity obtained when performing 4 × 4 min interval training session instead of the otherwise performed 3 × 8 min intervals. This figure shows (1) the numerically but not significantly higher training intensity performed by HYPER compared to NORM throughout the training intervention which was independent of the interval duration, (2) the increasingly higher training intensity performed by both groups during the intervention period which indicated that cycle performance was enhanced in both groups independent of hyperoxia.

Of the initial 32 trained cyclists, 23 of those (19 men and 4 women), with age 35.3 ± 6.4 years [mean ± standard deviation (SD)], body mass 75.2 ± 9.6 kg, height 179.8 ± 7.9 m, and VO_2_max of 4.5 ± 0.7 L·min^−1^, successfully adhered to the training regimen (NORM attended 95.8 ± 5.4% of the HIIT sessions and 90.1 ± 8.6% of the LOW1/LOW2 sessions; HYPER attended 94.4 ± 5.6% of the HIIT sessions and 95.5 ± 4.7% of the LOW1/LOW2 sessions) and were included for further analysis. The two groups NORM (*n* = 11) and HYPER (*n* = 12) did not differ at baseline for any measured variables. Participants’ characteristics are shown in Table 1. During the HIIT sessions, HYPER consistently trained at 3.3 ± 1.2% higher relative intensity than NORM despite a similar rating of perceived exertion (i.e., NORM RPE 8.3 ± 1.0 and HYPER RPE 8.2 ± 0.7). However, the higher training intensity did not lead to a significantly higher training load over the intervention compared to NORM (*p* = 0.37) (Figure 1).

**Table 1:** Characteristics and baseline measures of the hyperoxia (HYPER) and normoxia (NORM) group.

	NORM	HYPER
*n*	11	12
Women	1	3
Men	10	9
Age (years)	37.3 ± 4.5	33.4 ± 7.6
Height (cm)	182.8 ± 5.0	177.2 ± 8.6
Body mass (kg)	77.2 ± 7.5	73.2 ± 11.8
Time to exhaustion during maximal incremental test (s)	395.8 ± 55.3	406.3 ± 47.1
Winc. (W)	404.1 ± 45.0	387.9 ± 55.2
VO_2_max (L min^−1^)	4.7 ± 0.7	4.4 ± 0.7
VO_2_max (ml min^−1^ kg^−1^)	60.3 ± 5.1	60.4 ± 5.3
Mean power output during 20 min trial (W)	300.9 ± 41.5	281.0 ± 41.0
Mean power output during 20 min trial (W kg^−1^)	3.9 ± 0.4	3.9 ± 0.4
EIAH during 20 min trial (*n*)	7 (64%)	6 (50%)

Data are mean ± SD. Winc., maximum watt pedaled during the maximal incremental test; EIAH, exercise induced arterial hypoxemia (defined as SaO_2_ lower than 95%).

### Skeletal Muscle Mitochondrial Adaptations

**Figure 2:**
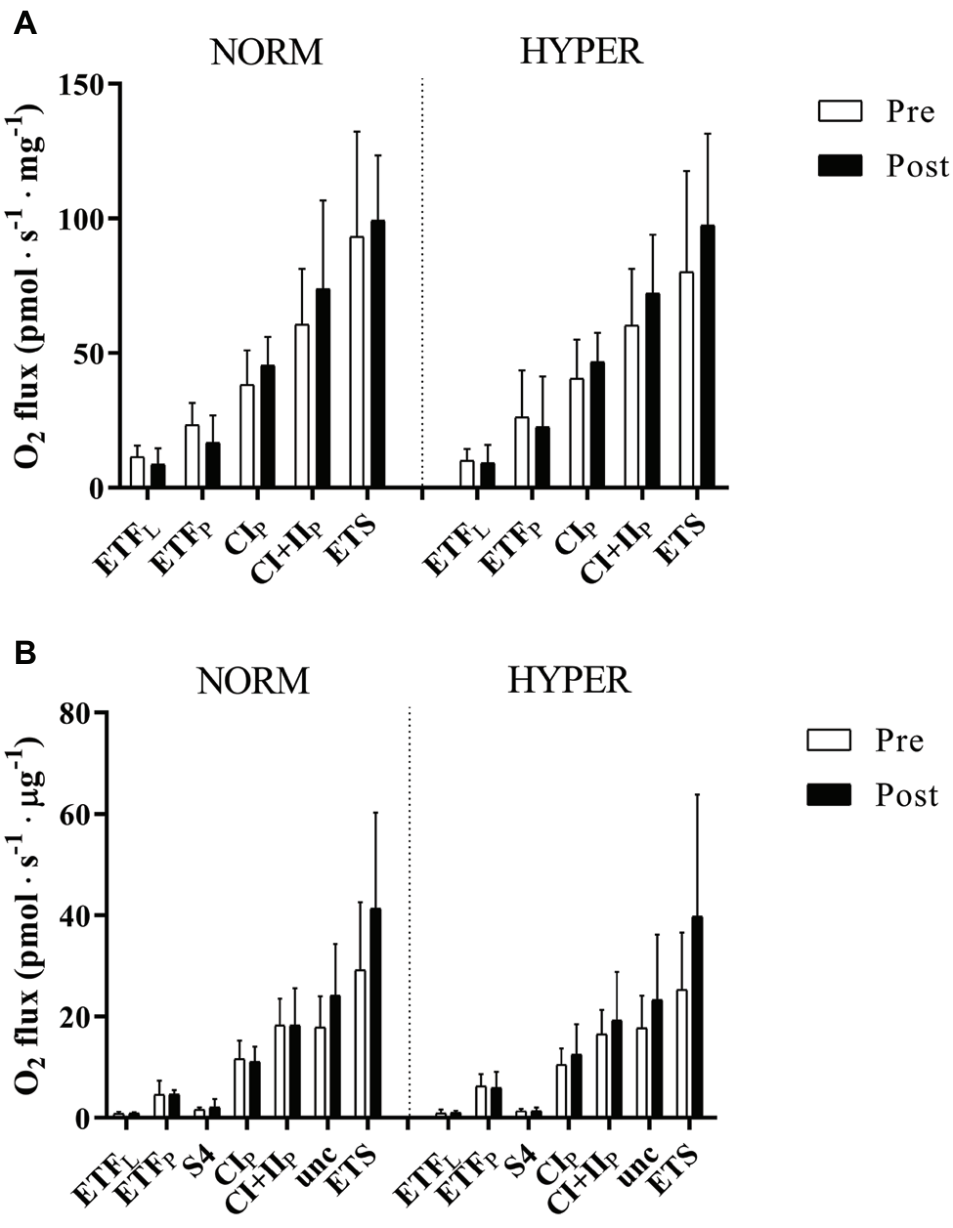
Mitochondrial respiration pre and post 6 weeks polarized and periodized high-intensity interval training either in normoxia (NORM) or hyperoxic-supplemented (HYPER). All values are expressed as means ± SD. In panel **(A)**, O_2_ flux rates obtained from measurement of mitochondrial respiration using permeabilized fibers technique are expressed in pmol s^−1^ mg^−1^ fibers initial wet weight, whereas in panel **(B)**, O_2_ flux obtained from measurement of isolated mitochondria the O_2_ flux rates are presented in pmol s^−1^ μg^−1^ protein. Electron-transferring flavoprotein complex (ETF), state 4 (S4), complex I (CI), complex I + II-linked substrate state (CI + II), and uncoupling state (Unc) electron transport system capacity (ETS). Leak respiration and oxidative phosphorylation are indicated by subscripts L and P, respectively.

**Figure 3:**
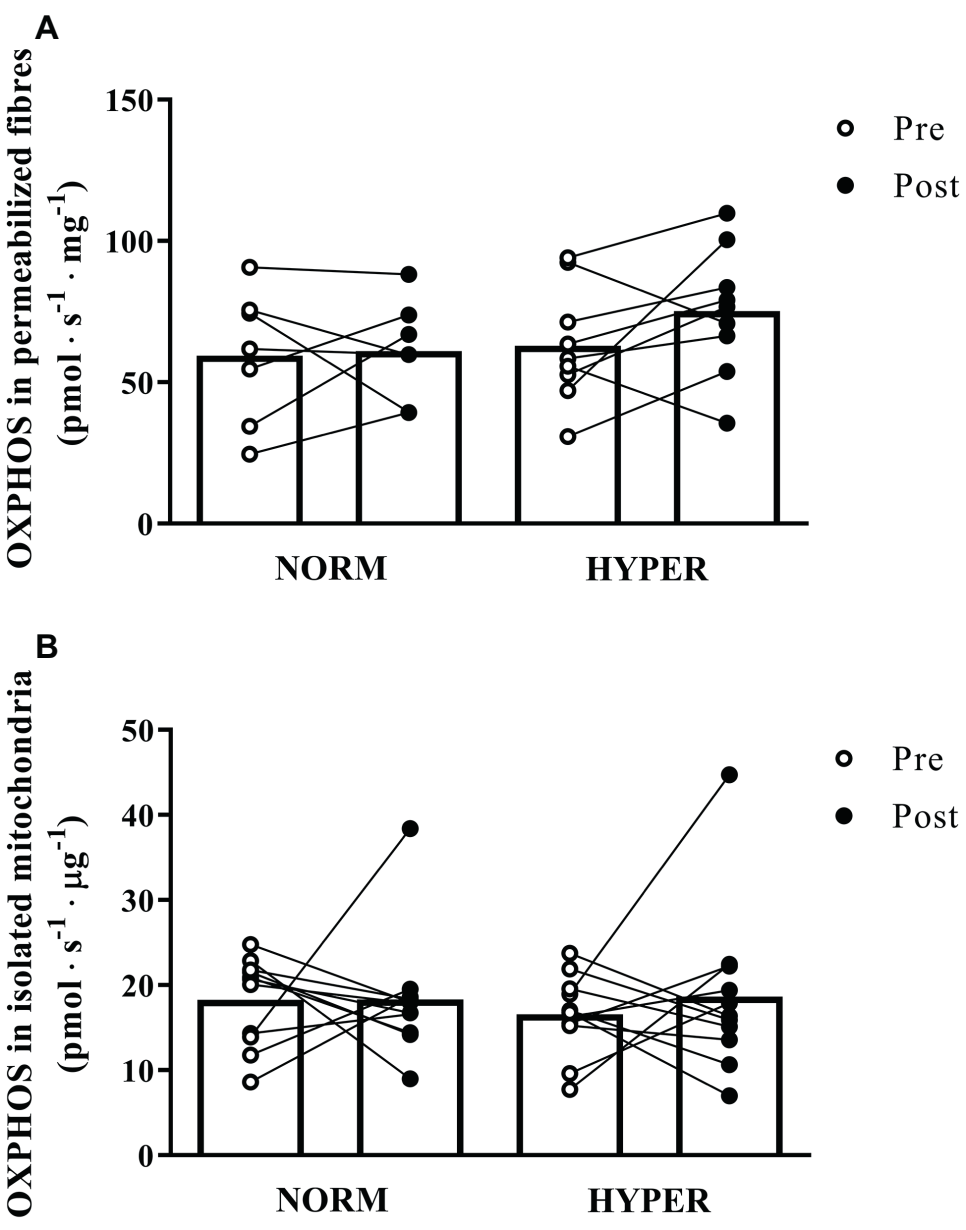
Maximal mitochondrial respiration (OXPHOS) pre and post 6 weeks polarized and periodized high-intensity interval training either in normoxia (NORM) or hyperoxic-supplemented (HYPER). All values are shown as mean and individual response following the intervention. In panel **(A)**, O_2_ flux rates obtained from measurement of mitochondrial respiration using permeabilized fibers technique are expressed in pmol s^−1^ mg^−1^ fibers initial wet weight, whereas in panel **(B)**, O_2_ flux obtained from measurement of isolated mitochondria are presented in pmol s^−1^ μg^−1^ protein.

The permeabilized fibers assay revealed that the training intervention had no effect on mass-specific (normalized to tissue wet weight) leak respiration, fatty acid oxidation, maximal oxidative phosphorylation respiratory, and electron transfer system capacity of the skeletal muscle with no difference between groups (Figure 2A). Maximal oxidative phosphorylation numerically increased (22.6 ± 46.1%) but did not reach the level of significance over the intervention (*p* = 0.20), between (*p* = 0.37, ES = 3.24) or within groups (NORM 16.5 ± 49.1% *p* = 0.86; HYPER 27.3 ± 46.0%, *p* = 0.15) and showed a great individual variability in response to exercise training (Figure 3). Although no differences over the intervention were detected between groups, fatty acid oxidation decreased over the intervention only in NORM (*p* = 0.03).

Similarly, the isolated mitochondria assay showed only a small relative increase (21.0 ± 75.1% *p* = 0.90) in intrinsic maximal mitochondrial respiration over the intervention (Figure 2B) with no change between (*p* = 0.66, ES = 0.51) or within groups (NORM 15.9 ± 73.3% *p* = 0.99; HYPER 26.1 ± 80.1%, *p* = 0.54).

The unchanged *ex vivo* mitochondrial oxidative phosphorylation over the intervention paralleled the unchanged biomarkers of mitochondrial content, i.e., citrate synthase (CS) activity (*p* = 0.42 and between groups *p* = 0.41). CS activity in NORM went from 238.7 ± 53.2 to 239.3 ± 39.5 nM min^−1^ mg^−1^ (*p* = 0.97) and HYPER from 239.4 ± 37.3 to 221.9 ± 48.6 nM min^−1^ mg^−1^ (*p* = 0.28). Similarly, cytochrome C oxidase protein levels did not change over the intervention (*p* = 0.93) and between groups (*p* = 0.95). Cytochrome C oxidase protein levels in NORM went from 11.6 ± 6.4 to 11.6 ± 4.2 a.u. (*p* = 0.99) and HYPER from 7.7 ± 3.6 to 7.6 ± 3.6 a.u. (*p* = 0.90).

### Hematology

Hematology variables are presented in Table 2. All parameters were unaltered by the training intervention and no differences were detected between groups.

**Table 2:** Body mass and intravascular volumes before and after training intervention.

	NORM		HYPER		E.S.	*p*
	Pre	Post	Pre	Post		
[Hb] (g L^−1^)	14.8 ± 0.7	14.8 ± 042	14.9 ± 1.1	14.4 ± 0.7	−1.06	0.56
Hct (%)	44.4 ± 2.2	45.3 ± 1.8	45.9 ± 3.0	44.7 ± 1.7	−1.87	0.31
*n*Hb (g)	962.6 ± 131.2	964.8 ± 160.7	986.4 ± 255.2	978.5 ± 292.8	−0.36	0.82
Hb_mass_ (g kg^−1^)	12.3 ± 0.5	12.3 ± 0.9	13.3 ± 1.7	13.0 ± 1.9	−0.16	0.92
RCV (ml)	2,889.9 ± 423.1	2,958.1 ± 516.6	3,038.6 ± 748.9	3,026.5 ± 891.8	−0.12	0.94
PV (ml)	3,650.0 ± 690.8	3,562.3 ± 520.1	3,547.7 ± 679.2	3,732.3 ± 1,060.7	1.11	0.22
BV (ml)	6,539.8 ± 1,097.5	6,520.4 ± 1,016.1	6,586.7 ± 1,385.0	6,758.7 ± 1,941.9	0.88	0.37

Values are means ± SD. [Hb], hemoglobin concentration; Hct, hematocrit; Hb_mass_, total hemoglobin mass; RCV, total red blood cell volume; PV, plasma volume; BV; blood volume. Measurements were performed before (Pre) and after Post training intervention in the group breathing normoxia (NORM: *n* = 6) and in the hyperoxia group (HYPER: *n* = 6). The level of significance and the effect size (E.S.) is indicated.

### Submaximal Measurements

Power outputs at lactate inflection point did not change over the intervention (*p* = 0.73). NORM went from a power output at lactate inflection point of 281.8 ± 42.1 to 282.9 ± 44.9 W (*p* = 0.78) and HYPER from 288.2 ± 37.2 to 289.3 ± 29.2 W (*p* = 0.84). No change in gross efficiency (GE) and work efficiency (WE) when cycling at 150 W were observed pre to post intervention with no difference between groups (NORM GE from 19.0 ± 0.9 to 19.3 ± 1.1% and HYPER GE 19.7 ± 0.1 to 20.0 ± 1.3%; NORM WE from 24.2 ± 1.0 to 24.9 ± 0.5% and HYPER WE from 25.0 ± 1.3 to 25.7 ± 1.7%).

### VO_2_max and time to exhaustion

Overall, VO_2_max was maintained over the course of the intervention (*p* = 0.58) with no difference between groups (*p* = 0.55, ES = 0.08). NORM went from a VO_2_max of 4.7 ± 0.7 to 4.6 ± 0.7 L min^−1^ (0.0 ± 3.7%, *p* = 0.84) and HYPER from 4.4 ± 0.7 to 4.5 ± 0.8 L min^−1^ (1.1 ± 3.8%, *p* = 0.33). Time to exhaustion significantly improved pre to post intervention from 401.3 ± 42.1 to 426.8 ± 49.7 s (*p* = 0.001) with no difference between groups (*p* = 0.31, ES = −0.18).

### Endurance cycling performance

**Figure 4:**
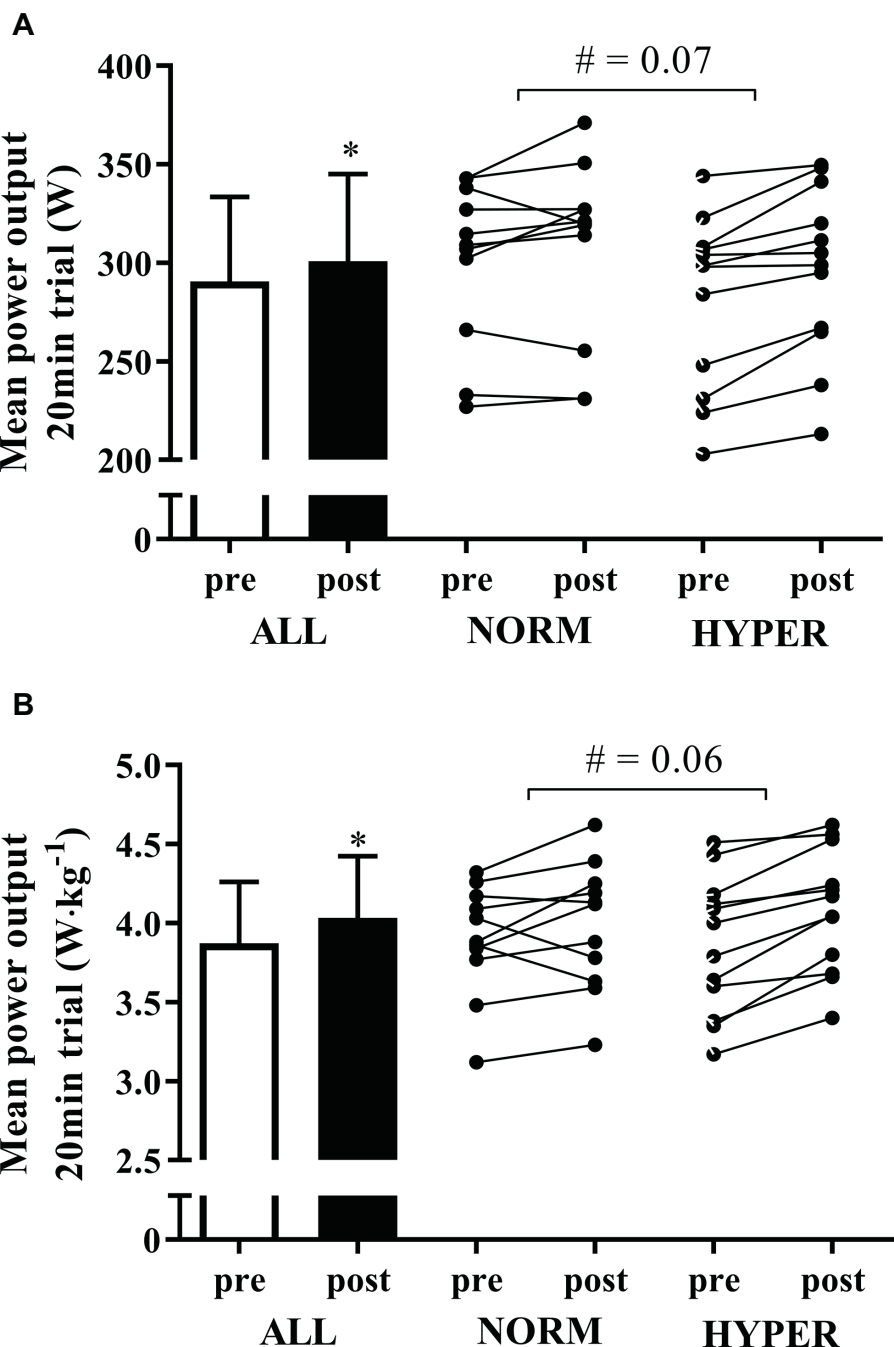
Mean ± SD of the mean power output during the self-paced 20-min cycling trial pre and post intervention expressed in absolute Watts **(A)** and Watts relative to body mass **(B)** for all the participants as well as the individual response of the participants in normoxia (NORM) and hyperoxia (HYPER) groups. Note: ^*^p < 0.05, ^#^*p* between groups.

Overall, the 6-week training intervention enhanced the absolute (3.7 ± 4.6%; *p* = 0.001) and relative to body mass (4.3 ± 4.7%; *p* = 0.0002) mean power output during the self-paced 20-min cycling trial (Figure 4). The magnitude of improvement in mean power output during the 20-min cycling trial was larger in HYPER compared to NORM (for mean power output, *p* = 0.07, ES = 0.22; for mean power output relative to body mass, *p* = 0.06, ES = 0.32). NORM numerically increased absolute mean power output during the 20-min cycling trial by 1.6 ± 4.3% (*p* = 0.23), whereas HYPER significantly enhanced power output by 5.6 ± 4.2% (*p* = 0.0006) (Figure 4). The power output during the 20-min test normalized per body mass numerically increased by 2.4 ± 6.0% in NORM (*p* = 0.15) and significantly in HYPER by 6.0 ± 3.7% (*p* = 0.0001).

## Discussion

This study presents novel findings on performance effects and physiological responses to high-intensity interval training with hyperoxia in already trained cyclists. We showed that 6-week high-intensity interval training induced non-significant, but potentially meaningful performance gains without affecting VO_2_max, hematological parameters, mitochondrial oxidative phosphorylation capacity, and biomarkers of mitochondrial content in already trained cyclists. Our results are in line with the study of Kilding et al. (2012) who, using a similar polarized training (Seiler, 2010) intervention with a F_I_O_2_ of 0.60, showed that training with hyperoxia had no significant physiological benefit in trained cyclists of a similar performance level to those tested in our study. This study shows that training with hyperoxia induces no change in hematological or muscle oxidative metabolic capacity as underlying mechanisms for endurance performance.

Hyperoxia acutely enables a higher exercise intensity and therefore larger mechanical work produced over the intervention compared to when breathing normoxia (Perry et al., 2005, 2007; Kilding et al., 2012). Despite a numerically greater exercise intensity in HYPER compared to NORM, contrary to our hypothesis, the higher relative intensity and greater mechanical work led to a small positive effect in performance compared to normoxia but did not induce superior physiological training adaptations. Of note, cyclists could only see cadence data during the HIIT sessions and were blind to breathing assignment, power output and heart rate while pedaling at the maximum effort during the 4- and 8-min intervals. Our findings indicate that a further increase in exercise intensity of an already high-intense exercise regimen does not necessarily lead to additional gains in skeletal muscle training adaptations. However, individual performance change over the intervention (Figure 4) revealed that breathing hyperoxia allowed a positive training response compared to normoxia in some individuals (2 of 11 cyclists in NORM slightly decreased power output during the 20-min cycling trial pre- to postintervention). We speculate that hyperoxic-supplemented training may be advantageous in individuals who show a lower magnitude of performance change compared to the group response. With this consideration, hyperoxic-supplemented endurance high-intensity training may have an effect in erroneously categorized “non-responders” prior to increasing training frequency as previously suggested (Montero and Lundby, 2017).

**Figure 5:**
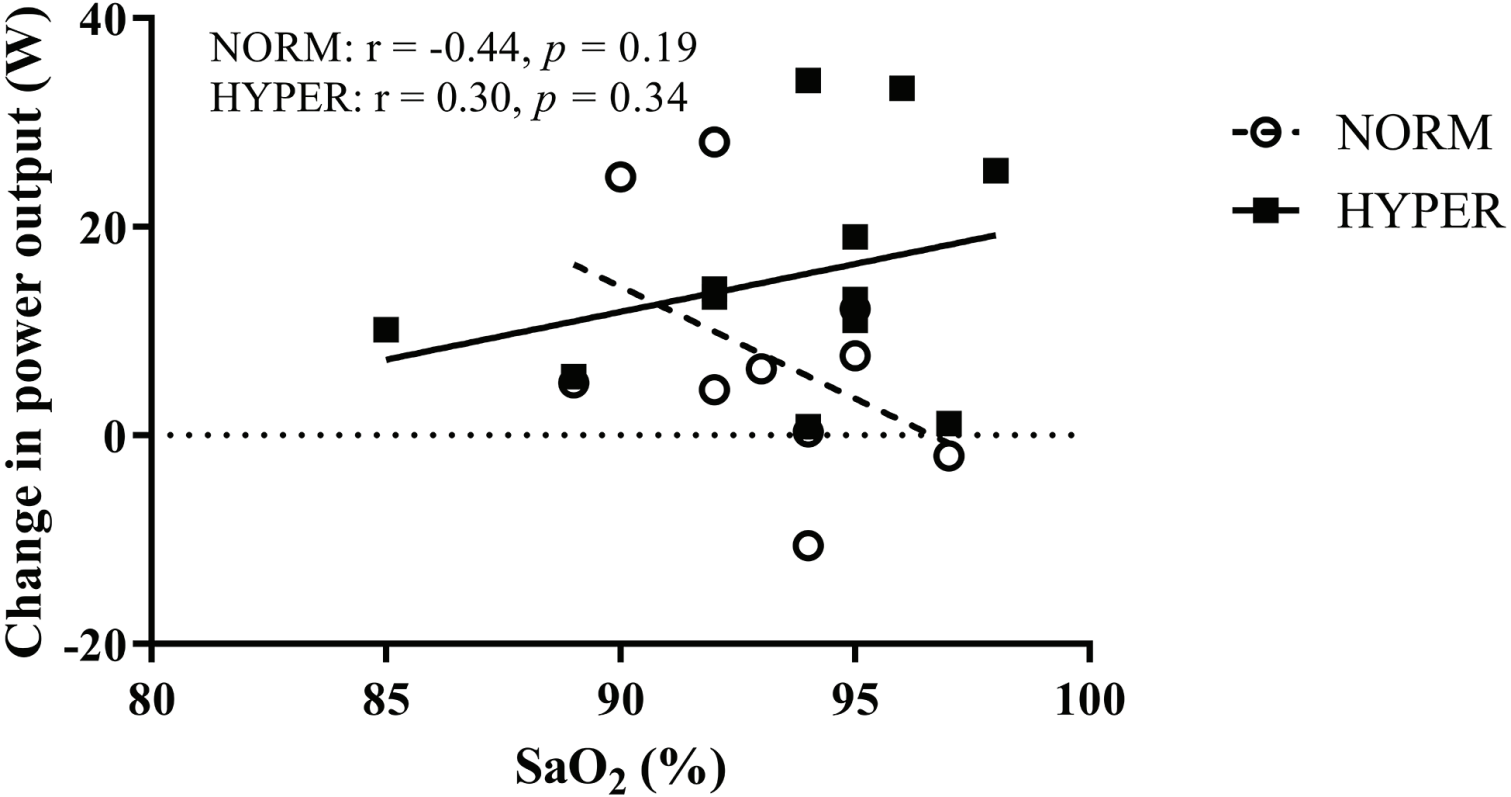
Relationship between the change in power output during the 20-min self-paced cycling test over the training intervention and the mean arterial oxygen saturation (SaO_2_) measured during the same test in participants in normoxia (NORM) and hyperoxia (HYPER) groups.

Improved arterial oxygen saturation with hyperoxia has been reported at exercise intensities near to VO_2_max thus preventing EIAH (Nielsen, 2003). EIAH is linked to the alveolar-capillary diffusion limitation due to decreased Hb mean-transit time in the lung (Dempsey and Wagner, 1999) caused by mechanical ventilatory constraint during exercise (Dominelli et al., 2013). EIAH has been reported to occur in ~50% of highly trained endurance athletes at sea level (Powers et al., 1988) and is more pronounced in both active and well-trained females (St Croix et al., 1998; Guenette and Sheel, 2007) than in male athletes (Anselme et al., 1994). In line with previous findings, 57% of our participants showed EIAH at intensities near to maximum; 7 of the 11 cyclists in the NORM, and 6 of the 12 cyclists in HYPER. The lower the SaO_2_ during exercise intensity near to maximum the larger is the effect of acutely breathing hyperoxia on exercise tolerance (Ohya et al., 2016). Therefore, it can be expected that maintaining Hb fully saturated at an exercise intensity near to VO_2_max in cyclists who otherwise exhibit EIAH in normoxia would lead to improved skeletal muscle training adaptations resulting from the increased O_2_ delivery. Contrary to what we hypothesized, increasing O_2_ delivery, partially preventing EIAH to occur, did not improve exercise adaptations when breathing hyperoxia. Furthermore, in HYPER, the change in performance did not correlate with the SaO_2_ levels during the cycle performance test (Figure 5). By contrast, there was a trend for an opposite relationship whereby cyclists manifesting high SaO_2_ during the cycle performance test in normoxia demonstrated a larger magnitude of change in performance over the hyperoxic intervention.

Fundamental papers have shown that the convective component of the oxygen cascade limits VO_2_max (Ekblom et al., 1968; Saltin and Calbet, 2006) and that the maximal mitochondrial oxidative phosphorylation is in excess over the O_2_ delivery (Boushel et al., 2011) in healthy individuals. We hypothesized that increasing microvasculature PO_2_ by hyperoxia would increase *in vivo* mitochondrial relative activation (Cardinale et al., 2018b), such that each mitochondrion would respire at a higher rate *in vivo* and in turn be exposed to a higher adaptive training stimulus. This hypothesis is supported by the greatly enhanced peripheral adaptations of skeletal muscle following one-legged cycling where higher O_2_ delivery per active muscle mass occurs compared to cycling (Abbiss et al., 2011). By contrast, we did not find support for this notion. It is likely that mitochondrial respiratory capacity may be still in excess of the O_2_ delivery despite the increased O_2_ carrying capacity due to hyperoxia while cycling. Our findings indicate that the greater mitochondrial activation induced when breathing hyperoxia compared to normoxia during cycling did not induce greater mitochondrial biogenesis and/or intrinsic function. It has been shown that hyperoxia does not increase *in vivo* mitochondrial respiration during exercise in obese untrained but only in patients with type II diabetes with impaired *ex vivo* mitochondrial respiration (Cree-Green et al., 2018). The unchanged *in vivo* mitochondrial respiration in obese untrained but overall healthy individuals when breathing hyperoxia can be explained by a lower mitochondrial O_2_ affinity (p50_mito_), a novel mechanism regulating oxygen diffusion from microvessels to muscle mitochondria with direct effects on oxygen consumption (Cardinale et al., 2018b). In the present study, the lack of change in the hematological variables that could have altered the O_2_ carry capacity and therefore O_2_ delivery over the intervention indicates that training with hyperoxia does not induce changes in the capacity for oxygen delivery or utilization.

The unchanged mitochondrial content pre to post intervention suggests either that our trained cyclists had already reached a mitochondrial content plateau prior to the intervention (Montero and Lundby, 2017), that the increase in O_2_ delivery with HYPER remained below mitochondrial capacity (maintained mitochondrial excess capacity) or that the training intervention did not increase the training volume to which our participants were accustomed to before the start of this study. The latter is supported by recent findings indicating that training volume significantly relates to CS activity (Granata et al., 2018) and that no plateau in CS activity should occur if training volume is constantly increased (Granata et al., 2018). However, the coefficient of variation of the change in OXPHOS measurements pre to post intervention (coefficient of variation of ~40%) was much larger than previously found in our laboratory (Cardinale et al., 2018a). The reason for this is unknown; we cannot discriminate if the variation came from the mitochondrial isolation procedure itself or if it was due to a large individual response to the hyperoxic and normoxic training stimulus. Nevertheless, the results on mitochondrial respiration should be interpreted with caution.

This study did not include direct neuromuscular measurements and it cannot be excluded that the numerically higher training intensity induced by hyperoxia increased muscle contractile properties which in turn led to small improvements in performance compared to normoxia. However, cycling efficiency was unchanged in both groups pre to post intervention. Furthermore, in Kilding et al., 2012’s study, hyperoxic training did not improve peak and mean power output during a 60-s cycle sprint compared to normoxia. Therefore, it is unlikely that our results are explained by improved neuromuscular properties post intervention in the hyperoxic group.

This study attempted to recreate the real training scenario of trained cyclists including (1) fluctuations of training intensities within the micro-cycle, (2) recovery days, and (3) a tapering period prior to post testing (Meeusen et al., 2013) in a periodized and polarized training intervention program while still maintaining the rigor of controlled trials with a parallel group study design. The significant improvement in performance in our participants indicates that the overall training intervention was successful.

In conclusion, this study showed that 6-week hyperoxic-supplemented high-intensity interval training produced a small, potentially meaningful effect on cycling performance. This response was not explained by cardiorespiratory, hematological, or mitochondrial factors measured in this study. The underlying mechanisms for the performance effects of hyperoxia training remain unexplained, and may raise ethical questions for elite sport.

## Data Availability

The datasets generated for this study can be found in figshare, https://figshare.com/s/90ea6a92bec059112c6b.

## Ethics Statement

This study was carried out in accordance with the recommendations of the Swedish Ethics Committee with written informed consent from all subjects. All subjects gave written informed consent in accordance with the Declaration of Helsinki. The protocol was approved by the Regional Ethical Review Board in Stockholm.

## Author Contributions

DC, RB, BE, FL, and PL contributed to the conception and design of the experiment. JL, TM, and OS supervised the training. DC, RB, FL, JL, TM, OS, and SM contributed to the data collection. DC analyzed and interpreted the data and wrote the first draft of the manuscript which was reviewed by the other co-authors. All authors read and approved the final manuscript.

### Conflict of Interest Statement

PL declares to have conflicts of interest and financial interest as co-founder of Oxelerate.

The remaining authors declare that the research was conducted in the absence of any commercial or financial relationships that could be construed as a potential conflict of interest.
